# Interaction of Cucurbit[7]uril with Oxime K027, Atropine, and Paraoxon: Risky or Advantageous Delivery System?

**DOI:** 10.3390/ijms21217883

**Published:** 2020-10-23

**Authors:** Jana Zdarova Karasova, Martin Mzik, Tomas Kucera, Zbynek Vecera, Jiri Kassa, Vit Sestak

**Affiliations:** 1 Department of Toxicology and Military Pharmacy, Faculty of Military Health Sciences, University of Defence in Brno, 50002 Hradec Kralove, Czech Republic; vecera.zbyna@gmail.com (Z.V.); jiri.kassa@unob.cz (J.K.); 2 Biomedical Research Center, University Hospital, 50002 Hradec Kralove, Czech Republic; 3 Department of Clinical Biochemistry and Diagnostics, University Hospital and Faculty of Medicine Hradec Kralove, 50002 Hradec Kralove, Czech Republic; martin.mzik@fnhk.cz (M.M.); vit.sestak@fnhk.cz (V.S.); 4 Department of Military Medical Service Organisation and Management, Faculty of Military Health Sciences, University of Defence in Brno, 50002 Hradec Kralove, Czech Republic; tomas.kucera2@unob.cz

**Keywords:** acetylcholinesterase, paraoxon, K027, mouse, pesticide, cucurbit[7]uril, CB7, cucurbiturils, antidote, in vivo

## Abstract

Antidotes against organophosphates often possess physicochemical properties that mitigate their passage across the blood–brain barrier. Cucurbit[7]urils may be successfully used as a drug delivery system for bisquaternary oximes and improve central nervous system targeting. The main aim of these studies was to elucidate the relationship between cucurbit[7]uril, oxime K027, atropine, and paraoxon to define potential risks or advantages of this delivery system in a complex in vivo system. For this reason, in silico (molecular docking combined with umbrella sampling simulation) and in vivo (UHPLC—pharmacokinetics, toxicokinetics; acetylcholinesterase reactivation and functional observatory battery) methods were used. Based on our results, cucurbit[7]urils affect multiple factors in organophosphates poisoning and its therapy by (i) scavenging paraoxon and preventing free fraction of this toxin from entering the brain, (ii) enhancing the availability of atropine in the central nervous system and by (iii) increasing oxime passage into the brain. In conclusion, using cucurbit[7]urils with oximes might positively impact the overall treatment effectiveness and the benefits can outweigh the potential risks.

## 1. Introduction

Antidotes against organophosphates (OPs, nerve agents, and pesticides) often possess physicochemical properties that mitigate their passage across the blood–brain barrier. In the past few decades, scientists have been developing drug delivery systems that would overcome the drugs’ non-optimal pharmacokinetic behavior and help them reach their pharmacodynamic targets. In the case of OPs poisoning, the bisquaternary reactivators (briefly named oximes) directly counteract the acetylcholinesterase (AChE, EC 3.1.1.7) inhibition [1,2] and atropine blocks muscarinic receptors [3,4]. The substantial hydrophilicity of quaternary moieties limits their distribution throughout the body (especially the brain), but these features are essential for the antidotal effect [5,6,7].

The development of novel carrier systems should fulfill three essential prerequisites: (i) improve drug bioavailability, (ii) increase targeting, and (iii) diminish a drug’s systemic toxicity. Cucurbit[7]urils (CB[7]) are pumpkin-shaped rigid macrocycles that can incorporate molecules into their cavity. CB[7] can bind positively charged aromatic compounds, platinum-based drugs with organic ligands, or specific metal complexes [8,9]. CB[7] has received significant attention as a potential drug delivery vehicle due to its excellent water solubility and cavity size fit for many commonly used drugs: coumarin—an anticoagulant [10], ranitidine—gastric ulcers treatment [11], atenolol—an antihypertensive, glibenclamide—an antidiabetic, memantine—Alzheimer’s disease treatment [12], pilocarpine—a parasympathomimetic [13], and cisplatin—an anti-cancer drug [14]; many of these results are from in vitro studies. Our previously published in vitro and in vivo results show that CB[7] can also form complexes with bisquaternary oximes [15,16]. The complexation facilitated the passage of oxime K027 through the blood–brain barrier. Total brain exposure (given as AUC_total_) of encapsulated K027 (K027@CB[7]) doubled compared to sole K027; encapsulated K027 also dwelled for longer in the brain [16]. Plumb and colleagues also reported that enveloping of drugs by CB[7] may improve peripheral organ targeting [17].

Despite extensive in vitro studies regarding the potential use of CB[7] for drug delivery and other biomedical application, clinically relevant in vivo results are still lacking. In vitro studies often investigate only one drug under stable conditions. Nonetheless, in vivo models are far from straightforward; the complexity increases with the presence of other xenobiotics, e.g., OPs and atropine in OPs poisonings.

In this study, we aimed to elucidate the relationships between oxime K027, atropine, paraoxon, and CB[7] and define the advantages and risks of this delivery system.

## 2. Results

### 2.1. In Silico Study

**Figure 1:**
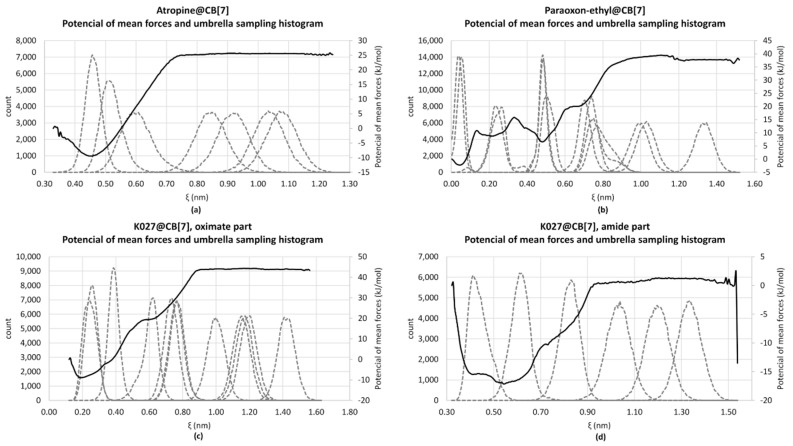
Potential of mean force (continuous line, right axis) and umbrella sampling histogram (dashed line, left axis) for the complexes (**a**) POX@CB[7], Gibbs free energy *Δ*G = −35.10 kJ/mol; (**b**) complex atropine@CB[7], Gibbs free energy *Δ*G = −41.72 kJ/mol; (**c**) K027@CB[7] where the oximate-part of K027 bound into CB[7], Gibbs free energy *Δ*G = −53.26 kJ/mol; (**d**) K027@CB[7] where the amide-part of K027 bound into CB[7], Gibbs free energy *Δ*G = −18.46 kJ/mol.

We determined the binding affinity of atropine, K027, and paraoxon-ethyl into the cavity of CB[7] as molar Gibbs free energy *Δ*G. We calculated the dissociation constants log K_D_ of the complex atropine@CB[7] (log K_D_ = 7.03), K027@CB[7] (log K_D_ = 3.11 for the amide-part and log K_D_ = 8.97 for the oximeter-part of the molecule), and paraoxon-ethyl@CB[7] (log K_D_ = 5.91). The potentials of mean forces and umbrella sampling histograms are shown in Figure 1.

### 2.2. LC-HRMS Analysis and Method Validation

**Figure 2:**
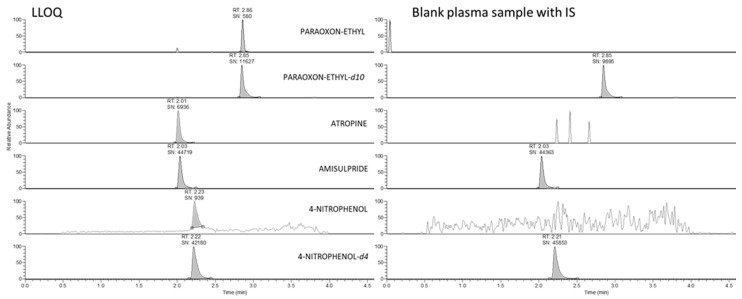
Comparison of LLOQ and blank plasma sample.

High-resolution mass spectrometer Q-Exactive focus with a combination of the UHPLC system allowed specific and sensitive measurement of all analytes as molecular ions with no need for fragmentation. Figure 2 documents the lack of interfering signals from the plasma matrix, confirming the method’s selectivity. Method sensitivity allowed the use of protein precipitation (PP) as the sample pre-treatment, which was convenient for two main reasons: (i) paraoxon instability in plasma or brain homogenate, which is rapidly enzymatically degraded to 4-nitrophenol. PP allowed rapid enzyme inactivation immediately after thawing plasma samples, or brain homogenization, and (ii) extraction for all three compounds in one procedure.

According to the EMA and FDA guidelines on bioanalytical method validation—EMA 2011 and FDA 2018 [18,19], we carried out a full validation for plasma and partial validation for brain homogenate samples. Partial method validation consisted of evaluating linearity, the limit of detection, recovery, and matrix effects. All the evaluated parameters met the required criteria; for details see the Supplementary Material (Supplementary Tables S1–S4). Calibration range (with limit of detection; LOD) was 0.5–256 ng/mL for paraoxon-ethyl (LOD = 0.20 ng/mL), 2–1024 ng/mL for atropine (LOD = 0.02 ng/mL), and 0.25–128 ng/mL for 4-nitrophenol (LOD = 0.10 ng/mL) for both types of material. Calibration dependence was linear with using weighting analysis 1/X^2 in all three analytes in plasma and brain homogenate. The recovery of analytes was slightly lower in brain homogenate compared to plasma. Deuterated paraoxon-ethyl and amisulpride as an internal standard for atropine effectively normalized matrix effect, where ionization of paraoxon-ethyl and atropine was significantly suppressed in the time of elution. On the other hand, 4-nitrophenol showed no sign of ion suppression or enhancement. This is probably due to measurements in negative polarity.

### 2.3. Pharmacokinetic Study of Atropine—Absorption and Brain Distribution

**Figure 3:**
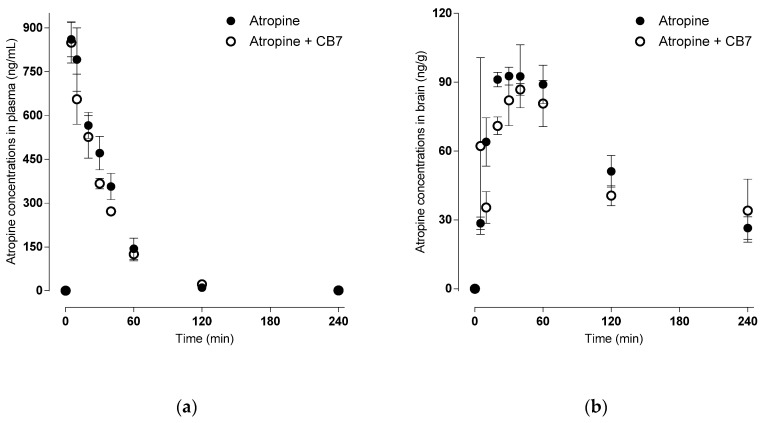
Levels of atropine were measured in mouse plasma (**a**) and brain (**b**) without CB[7] (ng/mL) (●), and with CB[7] (ng/mL) (○) collected at various time points up to 6 hr after a single i.m. bolus (10 mg/kg). Levels were measured in the presence of POX.

Pharmacokinetics of atropine seems to be unaffected by its encapsulation into CB[7], as can be documented by Figure 3 and Table 1. Atropine also appears to enter the brain quickly, with a more prolonged elimination half-live in the brain than plasma.

### 2.4. Toxicokinetics of Paraoxon versus Acetylcholinesterase Inhibition

Paraoxon-ethyl is rapidly metabolized to 4-nitrophenol mainly by paraoxonase (EC 3.1.8.1) [20], present primarily in plasma but also in tissues. Collection tube additives such as EDTA inhibit paraoxonase activity but paraoxon is also degraded through albumin [21], which is not influenced by EDTA. From after sample collection until freezing or protein precipitation, paraoxon degradation continues; it is not possible to distinguish between intravitally and artificially created 4-nitrophenol. Thus, all results related to paraoxon-ethyl and 4-nitrophenol plasma or brain homogenate concentration are expressed as the sum of both recalculated as the paraoxon equivalent.

**Figure 4:**
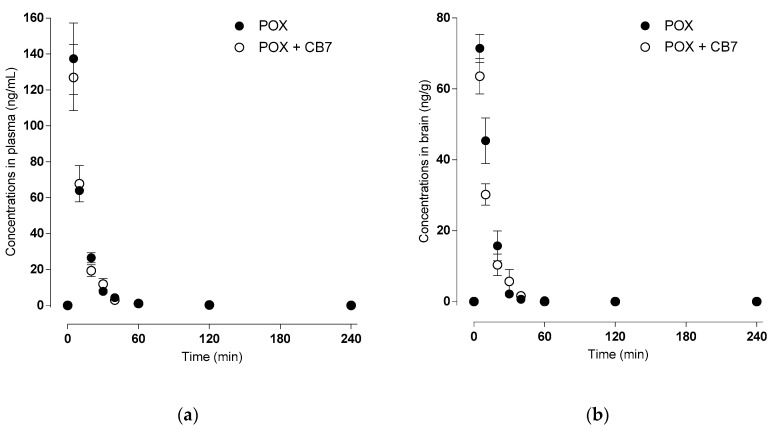
Levels of POX were measured in mouse plasma (**a**) and brain (**b**) without CB[7] (ng/mL) (●), and with CB[7] (ng/mL) (○) collected at various time points up to 6 hr after a single i.m. bolus (516 µg/kg). All animals were treated with atropine.

From these measured data, we deducted that paraoxon and nitrophenol are readily absorbed from the site of application, entering the brain rapidly with subsequent fast deposition from the brain and plasma. For details, see Figure 4 and Table 2.

**Figure 5:**
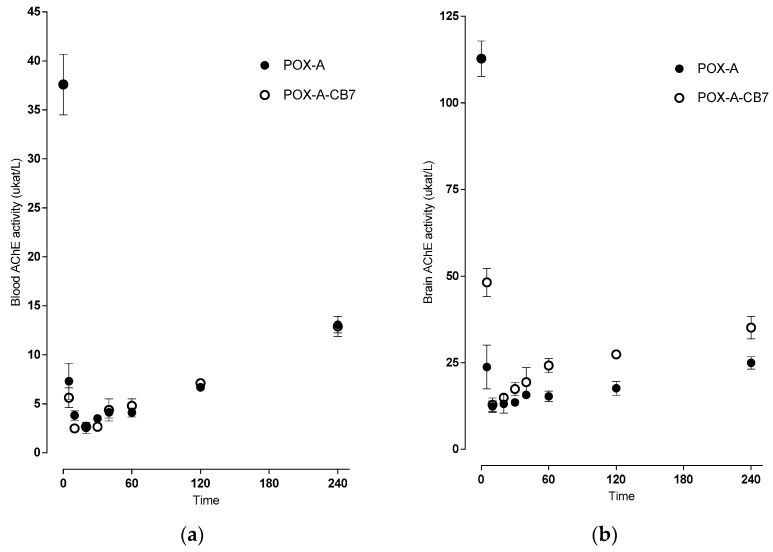
Changes in acetylcholinesterase activity (µkat/L) after dose equivalent to LD50 paraoxon administration (516 µg/kg, i.m.) measured in mouse blood (**a**) and brain (**b**) without CB[7], (●) and with CB[7] (○). Data were collected at various time points up to 6 hr (*n* = 3). All animals were treated with atropine (A).

Also, the acetylcholinesterase was quickly inhibited in both the blood and the brain. Its activity repeatedly rose with a significant difference upon administration of POX encapsulated in CB[7] in the brain compared to the standard administration (see Figure 5).

### 2.5. Pharmacodynamic Effectiveness of K027—Reactivation Study

**Figure 6:**
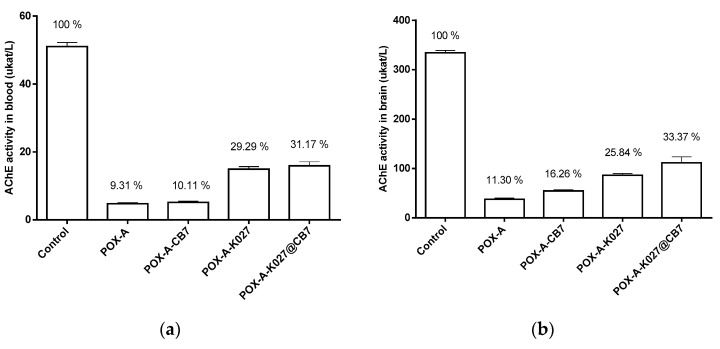
Changes in acetylcholinesterase activity (µkat/L) 60 min after LD50 paraoxon administration (516 µg/kg, i.m.) measured in mouse plasma (**a**) and brain (**b**) (*n* = 8).

The oxime K027 was able to significantly increase the activity of paraoxon-inhibited AChE in plasma as well as in the brain. While CB7 does not influence the reactivating efficacy of K027 in blood, the combination of the oxime K027 with CB7 brings markedly higher reactivation of paraoxon-inhibited AChE in the brain in comparison to K027 administered solely. The overview is in Figure 6.

### 2.6. Functional Observatory Battery

**Figure 7:**
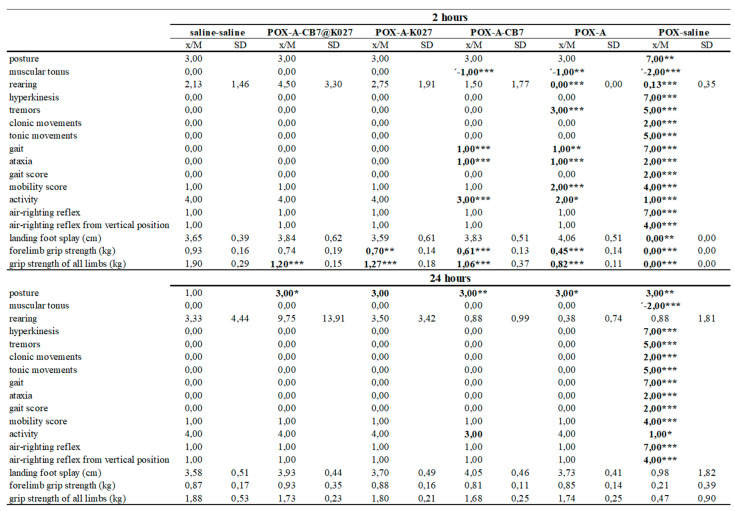
POX- influenced activity and neuromuscular neurotoxic markers deduced at 2 and 24 h after POX and treatment administration (*n* = 8). The difference was considered significant when *p* < 0.05 (*), *p* < 0.01 (**), or *p* < 0.005 (***).

**Figure 8:**
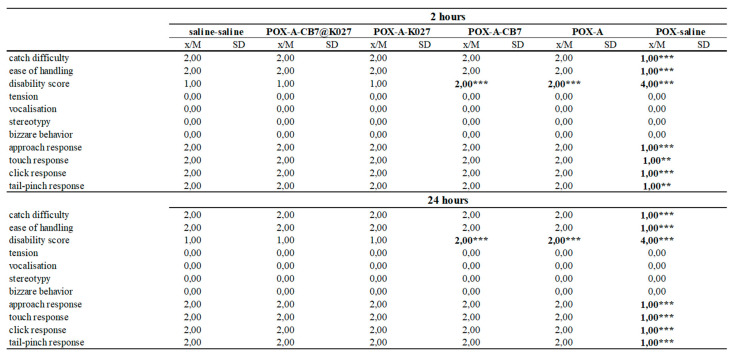
POX-influenced sensorimotor and neurotoxic excitability markers deduced at 2 and 24 h after POX and treatment administration (*n* = 8). The difference was considered significant when *p* < 0.05 (*), *p* < 0.01 (**), or *p* < 0.005 (***).

**Figure 9:**
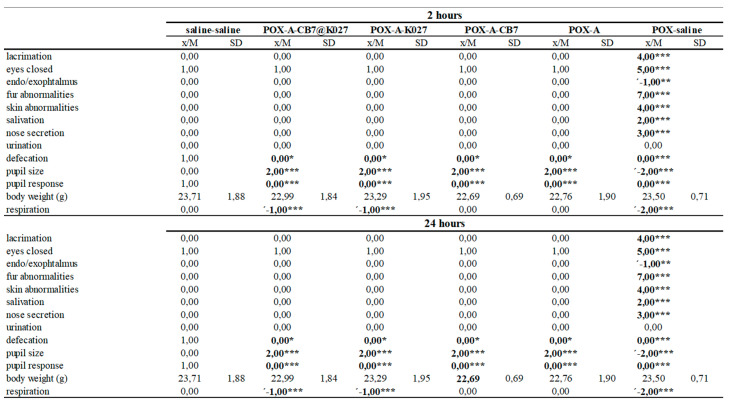
POX-influenced autonomic neurotoxic markers deduced at 2 and 24 h after POX and treatment administration (*n* = 8). The difference was considered significant when *p* < 0.05 (*), *p* < 0.01 (**), or *p* < 0.005 (***).

All paraoxon-poisoned mice survived until the end of the experiment (24 h) when the atropine alone, K027 or K027@CB[7] with atropine was used as treatment. Five non-treated mice died within 2 h of paraoxon administration. The behavioral results were divided into three parts (activity and neuromuscular, sensorimotor, and excitability and autonomic measures) [22]. The results overview is summarized in Figure 7, Figure 8 and Figure 9, and the overview/legend of functional observatory battery (FOB) scoring is found in the Supplementary Materials (Supplementary Table S5)

In overview, paraoxon induced many neurotoxic signs and symptoms indicated intense functional disorders in mice during the evaluated period. Paraoxon caused passive behavior and a wide range of symptoms resulting from the impaired neuromuscular transmission. No responses to stimuli were observed such as other typical signs of intoxication: miosis, lacrimation, salivation, and nose secretion. However, the administration of atropine alone was able to counteract some symptoms of paraoxon toxicity; the combination with CB[7] improves some other rated symptoms. This improvement was most pronounced in symptoms regarding activity and neuromuscular neurotoxicity (see Figure 7, 2 h). Further progress was observed after administration of K027, and K027@CB[7], respectively. From the other symptoms, the persistent effect of atropine on eye function in all groups or changes in respiration induced by the application of K027 was observed.

## 3. Discussion

The shift from in vitro to in vivo is often challenging; knowing CB[7] safety and mechanism of its actions is crucial before taking this step. In our studies, the specific drug–drug (CB[7], atropine, oxime K027, and paraoxon) interaction was evaluated. Both antidotes/OP may have some potential to be bound to the CB[7] cavity.

First, the binding of the paraoxon, atropine, and oxime K027 was proved by the computational methods. The molecular dynamics simulations showed the approximately similar binding affinity of atropine and paraoxon-ethyl—the binding energy (Gibbs free energy) of complex K027@CB[7] is roughly a half. The calculated dissociation constants of K027 differ from the experimental value log K_D_ = 6.72 [15]; they are between the calculated values for the amide- and the oximeter-part of the K027 molecule. The encapsulation of K027 into CB[7] may be a combination of both possibilities.

Our results correspond with the published data about the binding properties of cucurbiturils [23]. We observed the binding of positively charged parts of researched ligands. The positively charged nitrogen atoms of K027 and the protonated nitrogen of atropine interact with the partially charged oxygen atoms of CB[7]. The binding effect of CB[7] for the lipophilic part of paraoxon-ethyl and atropine and the aromatic part of K027 could be explained by the replacement of the high-energy water molecules in the free CB[7] [24]. It is the differences in the filling of the CB capacity that correspond to the differences in the calculated binding energies of the ligands. For atropine and the oxime part of K027, we observe the complete filling of the cavity and replacement of all water molecules. In contrast, for the amide part of K027 and paraoxon-ethyl, not all water molecules were displaced and therefore they showed lower binding energy.

For a better understanding of binding and potential drug–drug interaction processes with CB[7] under physiological conditions, we performed in vivo studies. First, we determined the plasmatic concertation–time profile of atropine after single i.m. administration. Atropine—with a pK_a_ of 9.7—exists under physiological pH predominantly in an ionized (protonated) form [25]. Protonation should lower the ability to cross biological barriers and enhance elimination via the kidney. Despite the high pK_a_ value, the increasing plasma concentration proves rapid absorption from the site of administration; the T_max_ was reached within 6.67 ± 1.36 min of administration. The atropine plasma level decreased rapidly after the completion of absorption (half-life 28.67 ± 0.93 min). Atropine is partially (i) destroyed by rodent specific enzyme (atropinase) that cleaves atropine, (ii) excreted without change via urine [26], and (iii) distributed throughout the body. The presence of CB[7] slightly changed all previously mentioned parameters: C_max_ was lower, T_max_ was reached earlier, and the half-life was shortened.

Atropine is an essential life-saving antidote for OPs intoxication [3,27]. Although it is generally accepted that atropine crosses the placental barrier [28], information about distribution across the blood–brain barrier is still scarce. The widely accepted hypothesis that atropine does little or does not act centrally has led to the introduction of new and sometimes more hazardous antidotes [29]. Our current study brings unique insight into the effect of atropine in the central nervous system. According to our results, the brain C_max_ of atropine reached more than a tenth of plasma concentration in 33.33 ± 2.72 min. A parameter that describes the brain distribution, the brain partition coefficient (Kp_Brain_, calculated as AUC_total-Brain_/AUC_total-Plasma_), reached 0.56; Kp_Brain_ increased with CB[7] to 0.71. This shift seems to be caused by slower elimination of atropine from the brain. The longer brain half-life and MRT support this assumption. Nonetheless, the mechanism by which CB[7] modulates atropine brain efflux remains unknown.

The cavity of CB[7] seems to bind some OPs, possibly changing OPs pharmacokinetics. Increasing brain targeting in such a way would be risky [30]. In the present study, we uncovered that CB[7] did not influence the T_max_ 5 min of paraoxon, but lowered C_max_: 137.41 ± 16.25 ng/mL without and 126.90 ± 15.11 ng/mL with CB[7]. The change in C_max_ (7.7%) might correspond to the proportion of paraoxon encapsulated in CB[7]. The inhibition of blood AChE was delayed; the minimal residual activity being 2.54 ± 0.57 µkat/L (6.8%) in 20 min, and 2.49 ± 0.16 µkat/L (6.6%) in 10 min, without and with CB[7], respectively. The AChE activity at later time points was comparable and increased gradually to 13.07 ± 0.85 µkat/L (34.8%) and 12.89 ± 1.03 µkat/L (34.3%), both in 240 min. The AChE activity might have regenerated by spontaneous reactivation and distribution and/or elimination of paraoxon from the organism; it is crucial that CB7 does not hinder these processes.

The comparison of peak brain paraoxon and paraoxon with CB[7] levels (71.44 ± 3.23 ng/g versus 63.57 ± 4.10 ng/g) and total brain exposure (expressed as AUC_total_, 899 ± 90 min.ng/g versus 723 ± 86 min.ng/g for paraoxon and paraoxon with CB[7], respectively) showed that CB[7] reduced the passage of paraoxon across the blood–brain barrier by 20%. Time-dependent differences in AChE activity were also evident: inhibition of brain AChE was comparable 12.36 ± 1.64 µkat/L (11.0%) and 12.86 ± 1.95 µkat/L (11.4%), without and with CB[7], respectively, both in 10 min but AChE regenerated faster in the presence of CB[7]. Our results are in good agreement with the study of Zhang and colleagues [31], where they used CB[7] as an oral antidote: CB[7] was able to bind paraquat in the gastrointestinal tract, thus inhibiting its absorption. The mechanisms in systemic circulation seem to be analogous.

As reported previously, the complexation enhances the passage of K027 into the brain [11]. Following animal protection laws, we performed only reactivation and behavioral study with K027 and paraoxon. In both cases, the CB[7] was administered in a dose equimolar to K027. CB[7] presence did not affect the AChE activity in blood, neither with atropine administered alone nor in combination with K027. Low AChE activity difference corresponds with results assessed previously (see Figure 5 blood in 60 min).

On the other hand, CB[7] with atropine alone was able to protect AChE in the brain by a further 5%. The brain AChE activity in animals treated with atropine, K027, and CB[7] was higher by 22% than those with atropine alone (see Figure 6). These surprising results may be caused by a combination of two CB[7] effects: (i) scavenging of paraoxon and (ii) increasing the K027 concentration in the brain. We assume that a small increase in brain AChE activity (10%) in certain parts of the brain correlates with the survival rates of intoxicated animals [32,33]. Restoration of the additional 10% AChE activity may influence potential sequelae, especially the neuropsychiatric and neurological impairments (memory, cognitive, mental, emotional, motor, and sensory), as described in survivors of organophosphate poisoning [34,35]. To confirm that, we carried out a behavioral study (functional observatory battery, FOB). In short, we can deduce that: (i) co-administration of CB[7] improves some signs of neurotoxicity, which was particularly noticeable in markers of activity and neurotoxic neuromuscular symptoms, and (ii) CB[7] did not worsen the prognosis of intoxication.

## 4. Materials and Methods

### 4.1. In Silico Prediction

Molecular docking, combined with an umbrella sampling simulation [36], was used to determine the binding affinity of paraoxon-ethyl, atropine, and K027 into CB[7]. Molecular docking calculations gained the initial pose of a ligand (paraoxon-ethyl, atropine, or K027). OpenBabel built the 3D structures of CB[7] and ligands, v. 2.3.2 [37], and optimized by Avogadro, v. 1.2.0-3 [38] using the force fields GAFF. They were converted into pdbqt-format by OpenBabel, v. 2.3.2. The docking calculations were done by Vina v. 1.1.2 [39]. We used two poses of K027—encapsulated amid- and oximate-parts of the molecule.

The force field parameters for CB[7] were gained from generalized Amber force field GAFF2 [40]. For ligands, they were generated by the software Antechamber, v. 20.0 [41]. The molecular dynamics simulation was carried out by Gromacs, v. 2020 [42]. The complex of CB[7] and ligand was solvated into a water box and neutralized by adding Na^+^ and Cl^−^ to a concentration of 10 nM. First, the system energy was minimized (maximum force < 1000.0 kJ/mol/nm), and the 100 ps isothermal-isochoric NVT and 100 ps isothermal-isobaric NPT equilibrations were done. Second, the pulling simulation was run along with the Z ax and frames extraction. The pulling parameters and frame spacing were set individually for each ligand. Each frame was prepared for simulation by 100 ps NPT equilibration, and the 10 ns simulation was done at a temperature of 310 K. The results were analyzed by the weighted histogram analysis method [43].

### 4.2. Animals

Babl/c mice weighing 22 ± 2 g were purchased from Velaz (Prague, Czech Republic). They were kept in an air-conditioned room with the light from 7:00 a.m. to 7:00 p.m. and were allowed access to standard food and tap water ad libitum. Mice were divided into groups of eight animals. The handling of the experimental animals was done under the supervision of the Ethics Committee of the Faculty of Military Health Sciences, Czech Republic.

### 4.3. Chemicals

Paraoxon-ethyl was purchased from Sigma-Aldrich (St. Louis, MO, USA). Oxime K027 (purities > 98%) was synthesized at the Department of Toxicology and Military Pharmacy of the Faculty of Military Health Sciences (University of Defense, Hradec Kralove). Paraoxon-ethyl-D10, 4-nitrophenol solution (5000 μg/mL in methanol), 4-nitrophenol-2,3,5,6-D4, atropine solution (1.0 mg/mL in acetonitrile), and amisulpride were purchased from Sigma-Aldrich (St. Louis, MO, USA). LC/MS grade acetonitrile, LC/MS grade methanol, LC grade chloroform, ammonium formate, and formic acid were purchased from Honeywell (Charlotte, NC, USA). All other drugs and chemicals of analytical grade were obtained commercially (Sigma-Aldrich) and were used without further purification. All substances were administered intramuscularly (i.m.) at a volume of 10 mL/kg b.w. to mice.

### 4.4. LC-HRMS Analysis

#### 4.4.1. LC-HRMS Parameters

All LC-MS analyses were performed on a Q-ExactiveTM Focus mass spectrometer equipped with a Heated-Electrospray Ionization II interface (HESI-II) (Thermo Scientific, San Jose, CA, USA) operating in positive (analysis of paraoxon-ethyl and atropine) or negative-ion mode (analysis of 4-nitrophenol) and UltimateTM 3000 RS pump Dionex (Thermo Scientific, San Jose, CA, USA) with a degasser, quaternary pump, cooled autosampler, and thermostat column compartment. Xcalibur software v. 4.0 (Thermo Scientific, San Jose, CA, USA) was used for data evaluation.

Critical parameters of mass spectrometer were set as follows: (1) for paraoxon-ethyl and atropine: positive polarity, spray voltage; +3.5 kV, S-lens RF level; +50 V, capillary temperature 290 °C, auxiliary gas heater temperature; 420 °C, sheet and auxiliary gas flow; 45 and 10 arbitrary units, respectively, (2) for 4-nitrophenol: negative polarity, spray voltage; +3.5 kV, S-lens RF level; +60 V, capillary temperature 270 °C, auxiliary gas heater temperature; 3800 °C, sheet and auxiliary gas flow; 45 and 10 arbitrary units, respectively. Data were acquired in full-scan MS mode (FullMS) at a resolution (m/Δm) of ~70,000 with the accuracy of measuring <2 ppm with quadrupole filter mass range 70–900 *m*/*z*. Paraoxon-ethyl, atropine, 4-nitrophenol, and internal standards were evaluated as molecular [M+H]^+^ or [M−H]^−^ ions, respectively. For exact *m*/*z* values and retention times of compounds, see Table 3.

Chromatographic separation was performed on Luna Omega 1.6 μm Polar C18, 50 × 2.1 mm ID (Phenomenex, Torrance, CA, USA), protected by a guard column SecurityGuard Ultra C18 (Phenomenex, Torrance, CA, USA). The separation was performed in gradient elution mode with a flow rate of 0.6 mL/min and following composition of mobile phase: (1) for paraoxon-ethyl and atropine (**A**) 0.1% FA in water (*v*/*v*) and (**B**) 0.1% FA in ACN/MeOH in ratio 50/50 (*v*/*v*), (2) for 4-nitrophenol (**A**) water and (**B**) ACN/MeOH in the ratio 50/50 (*v*/*v*). The gradient program was as follows: 0% **B** in 0–0.5 min, 0–100% B in 0.5–3.5 min, 100% **B** in 3.5–4.33 min, and column equilibration with 0% of **B** in 4.33–5.5 min. The total run time was 5.5 min. The autosampler and column oven temperatures were 10 and 45 °C, respectively. The sample injection volume was 10 μL for paraoxon-ethyl and atropine, 3.5 μL for 4-nitrophenol.

#### 4.4.2. Sample Preparation and Method Validation

Before analysis, the mouse brain (one hemisphere) was homogenized in distilled water (ratio 1:3 brain tissue/distilled water (*w*/*v*)) with a Lysing Matrix D homogenization tube (MP Biomedicals, Solon, USA) on the MagNA Lyser Instrument (Roche Diagnostics, Risch-Rotkreuz, Switzerland) for 10 s at 7000 rpm and centrifuged at 12,074× *g* for 5 min.

Two hundred microliters of brain homogenate or plasma were subjected to protein precipitation with 700 μL of ACN with internal standards. After vortexing (1 min, 1500 rpm) and centrifugation (5 min, 12,074× *g*), 50 μL were transferred to an injection vial and analyzed for 4-nitrophenol. The rest of the supernatant was transferred to a fresh, dry, and clean tube in a centrifuge-evaporator at 161× *g* at 60 °C for 40 min. The residue was dissolved in 100 μL 50% methanol/water (*v*/*v*), mixed for 1 min, transferred to an injection vial, and analyzed for paraoxon-ethyl and atropine.

Method validation (linearity, precision, accuracy, recovery, matrix effect, reproducibility, and stability) was performed for all analytes according to the Guideline on Bioanalytical Method Validation [13,14]. Corresponding blank material (mouse plasma and brain homogenate) could not be used directly—the spiked paraoxon is immediately enzymatically degraded to 4-nitrophenol [44]. Hence for the preparation of calibration and quality control (QC) samples, blank material was firstly precipitated by ACN with the content of IS. Subsequently, paraoxon was added.

### 4.5. Design of In Vivo Experiments

#### 4.5.1. Toxicokinetics of Paraoxon Versus Acetylcholinesterase Inhibition in the Blood and Brain—Pharmacokinetic Study of Atropine

The basic solution of paraoxon (1 mg/mL) was prepared in propylene glycol three days before starting the experiments. These basic solutions were diluted with saline (B Braun Medical, Germany) immediately before administration. Paraoxon-ethyl (516 µg/kg; LD50; 0.1 mL/10 g b.w., saline) was administered intramuscularly (i.m.). One minute later, animals received atropine alone (10 mg/kg; 0.1 mL/10 g b.w., saline) or atropine (10 mg/kg) in combination with CB[7] (17.7 mg/kg; 0.1 mL/10 g b.w., saline). Blood samples were collected from mice under deep terminal anesthesia by cardiac puncture into heparinized 1.5 mL tubes at 0, 5, 10, 20, 30, 40, 60, 120, and 240 min (*n* = 3). From each sample, 25 µL were taken for acetylcholinesterase activity assessment, and the rest was immediately centrifuged at 3000× *g* for 10 min (10 °C). Plasma samples were stored at −80 °C until LC-MS analysis (paraoxon and atropine).

The blood in the organs’ vessels also contains studied substances. This blood would interfere with the assay of exact brain tissue concentrations. Therefore, the animals were perfused transcardially with saline solution (0.9% NaCl) for 5 min (2 mL/min). After the wash-out, the skull was opened, and the brain was carefully removed; brains were stored at −80 °C until analysis.

#### 4.5.2. Dosing in Reactivation Study and Functional Observatory Battery

The basic solution of paraoxon (1 mg/mL) was prepared in propylene glycol three days before starting the experiments. These basic solutions were diluted with saline (B Braun Medical, Melsungen, Germany) immediately before administration. Paraoxon-ethyl (516 µg/kg; LD50; 0.1 mL/10 g b.w., saline) was administered intramuscularly (i.m.). One minute later, animals received atropine alone (10 mg/kg; 0.1 mL/10 g b.w., saline) or atropine (10 mg/kg) in combination with K027, or K027@CB[7]. The dose of oxime K027 was 29 mg/kg (i.m.) corresponds to 5% LD50 [45]. The dose of CB[7], either administered solely or as K027@CB[7], was equimolar to the dose of oxime K027 (i.m., 91.35 mg/kg) [16].

To evaluate the reactivating efficacy, mice were administered paraoxon i.m. at a dose, they were corresponding to LD50 (0.1 mL/10 g b.w., saline). One minute later, the animals received either atropine alone (10 mg/kg) or atropine (10 mg/kg) in combination with oxime K027, CB[7], or K027@CB[7]. The same design of administration was used in the functional observatory battery.

#### 4.5.3. Acetylcholinesterase Activity Assessment

Blood samples were collected under deep terminal anesthesia by cardiac puncture into heparinized 5 mL tubes at 60 min (*n* = 6). The blood was hemolyzed in Tris-HCl buffer (0.02 mol/L, pH 7.6, 1:20). The animals were perfused transcardially by saline solution (0.9% NaCl) for 5 min (10 mL/min) [5]. After perfusion, the brain was carefully removed and stored at 80 °C until the analysis. The brains were homogenized by the Ultra-Turrax T25 Basic homogenizer (IKA^®^-WERKE, Staufen, Germany) in Tris-HCl buffer (0.02 mol/L, pH 7.6, 1:10) to determine acetylcholinesterase (AChE) activity by a standard spectrophotometric method. Acetylthiocholine was used as a substrate (Tris-HCl buffer, 0.1 mol/L, pH 7.6). Helios Alpha spectrophotometer was used to determine the absorbance at 436 nm for blood and 412 nm for exsanguinated tissues. The AChE activity was derived from absorbance values with the calibration curve with cysteine and expressed as µkat/kg or µkat/L (µmol substrate hydrolyzed per kg wet tissue/L blood within 1 s) [46]. The values of the control group for blood, diaphragm, and brain AChE activity were obtained from rats administered with saline buffer (0.9% NaCl *w*/*v*) instead of the nerve agent and antidotes (saline control). The percentage of reactivation was calculated using the AChE activity values: {1 − [((saline control) − (oxime + atropine))/((saline control) − (atropine control))]} × 100 (modified equation by Clement et al.) [47].

#### 4.5.4. Functional Observatory Battery

Neuroprotective effects of atropine, K027, and K027@CB[7] were studied using a functional observatory battery (FOB). FOB is a standardized set of behavioral and neurophysiological observations, which was developed as a non-invasive procedure for detecting gross functional deficits related to the neurotoxic effects of studied compounds [22,48]. The FOB consists of measurements of sensory, motor, and autonomic nervous functions. The observer evaluates the posture, eyes, and involuntary motor movements. Each mouse is handled and observed in an open field test for 3 min. The exploratory activity, piloerection, and other skin abnormalities, salivation, nose secretion, gait characteristics as well as stereotypy and bizarre behavior, are noted and scored. Reflex testing includes a response to a frontal approach of the object, a direct touch, and the answer to an auditory stimulus. Bodyweight is also recorded. The study is blinded in such a way that the observer does not know the experimental design.

### 4.6. Data Evaluation

#### 4.6.1. Toxicokinetics of Paraoxon, Pharmacokinetics of Atropine

The time-dependent changes of paraoxon and atropine in each sample were recalculated to concentrations (µg/mL in plasma and µg/g in the brain) using the calibration curve, GraphPad Prism v. 6.05 (GraphPad Software, Inc., San Diego, CA, USA). The pharmacokinetic profile is calculated as mean ± SD (*n* = 6).

Standard no compartmental analysis was performed using the Kinetica software, v. 4.0 (InnaPhase Corporation, Thermo Fisher Scientific Inc., Waltham, MA, USA). The maximum concentration (C_max_) and the time to the maximum concentration (T_max_) were determined directly from the observed data. The area under the mean plasma concentration–time curve from zero to infinity (AUC_total_) was defined as the sum of the AUC_0–240 min_ and the extrapolated part, i.e., the ratio of the concentration predicted at the time interval of 24 h and the terminal rate constant λ_z_. The λ_z_ was estimated using linear regression of the log-transformed concentrations from 10 min to 4 h plotted against time. The half-life was calculated as follows: t_1/2_ = ln(2)/λz.

#### 4.6.2. Acetylcholinesterase Reactivations

The results variability was statistically evaluated by the standard deviation (SD) calculated for each group. The differences between groups were calculated using a one-way ANOVA test with Scheffe’s post hoc test. The differences were considered significant when *p* < 0.05.

#### 4.6.3. Functional Observatory Battery

Data collected with the FOB included categorical, ordinal, and continuous values. Statistical analyses were performed with a unique interactive program (NTX). The categorical and ordinal values were formulated as contingency tables and evaluated consecutively by the Chi-squared test of homogeneity, the concordance-discordance test, and the Kruskal–Wallis test. Successive statistical tests assessed the continuous data: CI for delta, the Bartlett test for equality of variance, the William test, and the test for distribution functions [49]. The differences were considered significant when *p* < 0.05.

## 5. Conclusions

In conclusion, the OPs cross the blood–brain barrier and inhibit AChE that can initiate progressive brain damage with neuronal dysfunction [50], but the current antidotes act predominantly in the periphery. To improve the protection of the central nervous system, we can exploit different strategies such as (i) use of scavengers or bio-scavengers to sequester OPs in the peripheral compartment [51], (ii) use of a centrally active anticholinergic drug [29], or (iii) increase in oxime brain targeting [16,52]. Based on our present results, CB[7] affects multiple factors OPs poisoning and its therapy by (i) scavenging paraoxon and preventing free fraction of this toxin from entering the brain, (ii) enhancing the availability of atropine in the central nervous system and by (iii) increasing oxime passage into the brain. Using CB[7] with oximes might positively affect the treatment effectiveness, and the benefits can outweigh the potential risks. To make a scientifically sound decision, mammalian toxicity of CB[7] and the mechanism of its protection/toxic action remain yet to be assessed.

## Figures and Tables

**Table 1:** Pharmacokinetic parameters obtained after a single i.m. injection of atropine (10 mg/kg). Values are given as mean ± SEM (*n* = 3).

PLASMA	Atropine	Atropine-CB[7]
C_max_ (ng/mL)	860.48 ± 48.74	849.17 ± 56.96
T_max_ (min)	6.67 ± 1.36	5.00 ± 0.00
AUC_total_ (min × ng/mL)	31,028 ± 2956	27,643 ± 2903
λz (L/min)	0.024 ± 0.01	0.027 ± 0.00
Half-life (min)	28.67 ± 0.93	25.96 ± 0.03
MRT (min)	31.25 ± 0.90	33.10 ± 2.08
CL (L/min/kg)	0.33 ± 0.03	0.37 ± 0.04
Vz (L/kg)	13.84 ± 1.80	13.97 ± 1.42
Vss (L/kg)	10.32 ± 0.96	12.12 ± 0.54
**BRAIN**		
C_max_ (ng/mL)	99.58 ± 8.43	107.28 ± 4.89
T_max_ (min)	33.33 ± 2.72	31.67 ± 13.00
AUC_total_ (min × ng/mL)	17,361 ± 2092	19,582 ± 4343
λz (L/min)	0.007 ± 0.001	0.006 ± 0.002
Half-life (min)	105.87 ± 8.71	153.66 ± 52.82
MRT (min)	164.53 ± 12.01	231.54 ± 72.24

Cmax = peak plasma concentration; Tmax = time to Cmax; AUCtotal = area under the concentration-time curve from zero to infinity; λz = terminal elimination rate constant; half-life refers to the elimination phase; MRT = mean residence time; CL = clearance; Vz = volume of distribution during terminal phase; Vss = apparent volume of distribution at steady state.

**Table 2:** Pharmacokinetic parameters obtained after a single i.m. administration of paraoxon (516 µg/kg). Values are given as mean ± SD (*n* = 3).

PLASMA	POX	POX-CB[7]
C_max_ (ng/mL)	137.41 ± 16.25	126.90 ± 15.11
T_max_ (min)	5.00 ± 0.00	5.00 ± 0.00
AUC_total_ (min × ng/mL)	1588 ± 144	1524 ±122
λz (L/min)	0.070 ± 0.002	0.063 ± 0.015
Half-life (min)	9.91 ± 0.26	14.75 ± 5.11
MRT (min)	12.47 ± 0.40	13.66 ± 0.71
CL (L/min/kg)	0.33 ± 0.03	0.34 ± 0.03
Vz (L/kg)	4.75 ± 0.39	7.39 ± 2.62
Vss (L/kg)	4.12 ± 0.26	4.72 ± 0.44
**BRAIN**		
C_max_ (ng/mL)	71.44 ± 3.23	63.57 ± 4.10
T_max_ (min)	5.00 ± 0.00	5.00 ± 0.00
AUC_total_ (min × ng/mL)	899 ± 90	723 ± 86
λz (L/min)	0.114 ± 0.029	0.103 ± 0.003
Half-life (min)	8.57 ± 3.20	6.76 ± 0.19
MRT (min)	11.35 ± 0.85	11.62 ± 0.77

Cmax = peak plasma concentration; Tmax = time to Cmax; AUCtotal= area under the concentration–time curve from zero to infinity; λz = terminal elimination rate constant; half-life refers to the elimination phase; MRT = mean residence time; CL = clearance; Vz = volume of distribution during terminal phase; Vss = apparent volume of distribution at steady state.

**Table 3:** The summary of the analytes.

Name	Molecular Formula	Theoretical Mass [M+H]^+^	Retention Time (min)
Paraoxon-ethyl	C_10_H_14_NO_6_P	276.06315	2.86
Paraoxon-ethyl-d10	C_10_H_4_D_10_NO_6_P	286.12592	2.85
Atropine	C_17_H_23_NO_3_	290.17507	2.01
Amisulpride	C_17_H_27_N_3_O_4_S	370.1795	2.03
**Name**	**Molecular Formula**	**Theoretical Mass [M+H^+^]^−^**	**Retention Time (min)**
4-Nitrophenol	C_6_H_5_NO_3_	138.01967	2.24
4-Nitrophenol-d4	C_6_HD_4_NO_3_	142.04477	2.23

## Supplementary Materials

The following are available online at https://www.mdpi.com/1422-0067/21/21/7883/s1.

## Abbreviations

A	Atropine
AChE	Acetylcholinesterase
AUC_total_	The area under the concentration–time curve from zero to infinity
C_max_	Peak plasma/brain concentration
CB[7]	Cucurbit[7]uril
xx@CB[7]	Compounds (xx—atropine, paraoxon, or oxime K027) encapsulated into cucurbit[7]uril
FOB	Functional observatory battery
OPs	Organophosphates—nerve agents and pesticides
PP	Protein precipitation
POX	Paraoxon
QC	Quality control
T_max_	Time to C_max_
