# Supplementary material for: Interaction of Cucurbit[7]uril with Oxime K027, Atropine, and Paraoxon: Risky or Advantageous Delivery System?
Source: Int J Mol Sci. 2020 Oct 23;21(21):7883. doi: 10.3390/ijms21217883 (PMC7672622; doi:10.3390/ijms21217883)
Supplement: Supplementary file 1 [file ijms-21-07883-s001.pdf]

Suplementary Material

Interaction of cucurbit[7]uril with oxime K027, atropine, and paraoxon: Is this delivery system risk or advantage?

Jana Zdarova Karasova [1,2,*], Martin Mzik [3], Tomas Kucera [4], Zbynek Vecera [1], Jiri Kassa [1] and Vit Sestak [3]

[1] Department of Toxicology and Military Pharmacy, Faculty of Military Health Sciences, University of Defence in Brno Hradec Kralove, Czech Republic; zdarova.jana@gmail.com
[2] Biomedical Research Center, University Hospital, Hradec Kralove, Czech Republic; zdarova.jana@gmail.com
[3] Department of Clinical Biochemistry and Diagnostics, University Hospital and Faculty of Medicine Hradec Kralove, Hradec Kralove, Czech Republic; vit.sestak@fnhk.cz
[4] Department of Military Medical Service Organisation and Management, Faculty of Military Health Sciences, University of Defence in Brno Hradec Kralove, Czech Republic; tomas.kucera2@unob.cz
* Correspondence: Assoc. Prof. Jana Zdarova Karasova, zdarova.jana@gmail.com; Tel.: +420-973-255-164

Supplementary Material

We carried out a full validation for plasma and partial validation for brain homogenate samples according to the EMA and FDA guidelines on Bioanalytical method validation - EMA 2011, FDA 2018. All the evaluated parameters met the required criteria; detailed validation results are presented in **Table 1-4.**

Table 5 is a legend for Functional observatory battery (FOB) scoring.

Table 1. Reproducibility of calibration curves (n=6 for plasma, n=4 for the brain)

		PARAOXON	4-NITROPHENOL	ATROPINE
PLASMA	Calibration model	Linear fit	Linear fit	Linear fit
	Weighting analysis	1/X^2	1/X^2	1/X^2
	Coefficient of determination R^2	>0.997	>0.997	>0.995
	Regression coefficient *a*	0,0174	0,0208	0,0167
	Standard deviation SD(*a*)	0,0015	0,0007	0,0010
	Relative standard deviation RSD(*a*)	8,7%	3,4%	5,9%
BRAIN	Calibration model	Linear fit	Linear fit	Linear fit
	Weighting analysis	1/X^2	1/X^2	1/X^2
	Coefficient of determination R^2	>0.996	>0.995	>0.997
	Regression coefficient *a*	0,0165	0,0219	0,0152
	Standard deviation SD(*a*)	0,0011	0,0012	0,0013
	Relative standard deviation RSD(*a*)	6,6%	5,4%	8,5%

Table 2. Summary of validation results (precision and accuracy) in mouse plasma for four different concentrations - limit of quantitation(LLOQ), low (QC A), mid (QC B) and high (QC C) level, and diluted level (QC D)

		PARAOXON					4-NITROPHENOL					ATROPINE				
		LLOQ	QC A	QC B	QC C	QC D	LLOQ	QC A	QC B	QC C	QC D	LLOQ	QC A	QC B	QC C	QC D
	ng/mL	0,50	1,25	20,80	192,00	19,20	0,50	1,25	20,80	192,00	19,20	2,50	5,00	83,20	768,00	76,80
PLASMA	**Within-batch 1**															
	Mean (ng/mL)	0,45	1,25	21,29	204,85	17,83	0,51	1,32	22,62	209,49	18,45	2,25	5,28	84,63	786,64	71,12
	Precision (%CV)	11,7%	8,3%	2,3%	2,5%	2,5%	7,1%	2,5%	0,8%	1,4%	2,6%	8,3%	6,1%	1,6%	4,3%	4,0%
	Accuracy (%)	-10,3%	0,1%	2,4%	6,7%	-7,2%	1,4%	5,8%	8,8%	9,1%	-3,9%	-9,9%	5,6%	1,7%	2,4%	-7,4%
	**Within-batch 2**															
	Mean (ng/mL)	0,48	1,22	20,79	205,93	18,37	0,52	1,28	22,22	205,89	18,66	2,31	5,26	80,94	702,16	71,23
	Precision (%CV)	10,4%	6,1%	1,7%	1,2%	1,3%	4,7%	0,9%	1,7%	1,9%	2,5%	8,3%	5,3%	2,8%	3,5%	3,8%
	Accuracy (%)	-3,5%	-2,3%	-0,1%	7,3%	-4,3%	4,4%	2,7%	6,8%	7,2%	-2,8%	-7,7%	5,2%	-2,7%	-8,6%	-7,2%
	**Within-batch 3**															
	Mean (ng/mL)	0,46	1,22	20,00	193,87	18,12	0,48	1,31	22,18	206,62	18,57	2,70	5,54	88,11	786,77	74,24
	Precision (%CV)	5,9%	2,2%	2,0%	1,4%	3,4%	4,1%	4,3%	1,3%	1,8%	1,7%	3,9%	3,0%	1,1%	2,9%	3,7%
	Accuracy (%)	-8,4%	-2,6%	-3,8%	1,0%	-5,6%	-4,1%	4,6%	6,6%	7,6%	-3,3%	8,2%	10,8%	5,9%	2,4%	-3,3%
	**Batch-to-batch**															
	Mean (ng/mL)	0,46	1,23	20,73	201,76	18,09	0,50	1,30	22,34	207,33	18,56	2,42	5,36	84,56	758,52	72,20
	Precision (%CV)	9,5%	5,8%	3,3%	3,2%	2,7%	6,2%	3,0%	1,5%	1,8%	2,2%	10,7%	5,2%	4,0%	6,4%	4,1%
	Accuracy (%)	-7,4%	-1,6%	-0,3%	5,1%	-5,8%	0,6%	4,4%	7,4%	8,0%	-3,3%	-3,1%	7,2%	1,6%	-1,2%	-6,0%

Table 3. Summary of matrix effect and recovery

		PARAOXON			4-NITROPHENOLE			ATROPINE		
		QC A	QC B	QC C	QC A	QC B	QC C	QC A	QC B	QC C
PLASM	ME % (no IS)	60,3 ± 3.6%	69.9 ± 2,1%	69,9 ± 4,0%	97.9 ± 5,7%	94.1 ± 3,4%	99.1 ± 3.2%	84.3 ± 2.2%	78.4 ± 6.7%	82.8 ± 2,9%
	ME %	90 ± 1,1%	99,1 ± 0,8%	99,9 ± 0,7%	109,9 ± 4,0%	100,4 ± 0,6%	96,4 ± 1,2%	92,5 ± 1,2%	88,9 ± 2,7%	94,9 ± 1,7%
	RE %	48,8 ± 4,8%	42,6 ± 1,2%	53,5 ± 1%	99.5 ± 3,7%	93,1 ± 1,6%	97,7 ± 0,9%	87,7 ± 1,3%	88,5 ± 1,6%	81,8 ± 0,8%
BRAIN	ME % (no IS)	54,3 ± 6,6%	58,8 ± 4,7%	61,0 ± 4,2%	95,8 ± 6,5%	96,1 ± 5,7%	92,1 ± 2,0%	75,4 ± 5,3%	73,2 ± 4,0%	78,8 ± 3,5%
	ME %	91,3 ± 3,3%	99,7 ± 0,9%	101,1 ± 0,5%	103,9 ± 3,6%	101,4 ± 1,5%	96,5 ± 0,8%	94,4 ± 1,5%	93,6 ± 1,1%	99,4 ± 1,5%
	RE %	42,1 ± 6,2%	45,9 ± 3,2%	48,2 ± 4,9%	94.2 ± 4,7%	95,8 ± 3,2%	90,2 ± 3,9%	80,4 ± 4,1%	85,8 ± 2,2%	79,9 ± 4,7%

ME % (no IS) - Matrix effect without IS normalization; ME % - Matrix effect normalized with IS ; RE % - Recovery

Table 4. Stability evaluation in mouse plasma as the average deviation from the reference value

		PARAOXON		4-NITROPHENOL		ATROPINE	
		QC B	QC C	QC B	QC C	QC B	QC C
	Autosampler stability	-2,6%	-2,5%	-1,4%	-1,5%	-3,9%	-7,0%
PLASMA	Freeze-Thaw stability	NE	NE	-4,9%	-4,3%	-2,5%	-4,7%
	Long-term stability	NE	NE	-8,6%	-6,4%	-10,5%	-5,6%

NE - not evaluated

Table 5. Legend for FOB scoring

Marker	Scored values									
	**-2**	**-1**	**0**	**1**	**2**	**3**	**4**	**5**	**6**	**7**
**posture**				***sitting or standing***	***rearing***	***asleep***	flattened	lying on side	crouched over	head bobbing
**catch difficulty**				passive	***normal***	defense	flight	escape	aggression	
**ease of handling**				very easy	***easy***	moderately difficult	difficult			
**muscular tonus**	atonic	hypotonic	***normal***	hypertonic	rigidity	fasciculation				
**lacrimation**			***none***	slight	severe	crusts	colored crusts			
**eye closed**				***open***	slightly dropping	half-way dropping	completely shut	ptosis		
**endo/exophthalmos**		endophtalmos	***normal***	exophthalmos						
**fur abnormalities**			***normal***	colored	disheveled	colored + disheveled	bald spot	injury	other changes	piloerection
**skin abnormalities**			***normal***	pale	erythema	cyanosis	pigmentation	cold	injury	
**salivation**			***none***	slight	severe					
**nose secretion**			***none***	slight	severe	colored				
**hyperkinesis (hyperkinetic disorders)**			***normal***	repetitive movements of mouth and jaws	non-rhythmic quivers	mild tremors	severe tremors	myoclonic jerks	clonic convulsion	
**tremors**			***none***	only during stimulation	mild/local	mild/overall	affecting movement	impeding movement		
**clonic movements**			***none***	twitches	non-rhythmic movement					
**tonic movements**			***normal***	contraction of extensors	opistotonus	emprostotonus	explosive jumps	tonic convulsions		
**gait**			***normal***	ataxia	overcompensat ion of hind limb movements	feet point outwards from the body	forelimbs are extended	walks on tiptoes	hunched body	the body is flattened against the surface
**ataxia (movement coordination disorder)**			***none***	mild	strong					
**paresis (disturbance of free movement)**			***none***	mild	strong					
**gait score**				***normal***	slightly impaired	somewhat impaired	impaired			
**mobility score**				***normal***	slightly impaired	somewhat impaired	impaired			
**activity**				very low	sporadic	reduced	***normal***	enhanced	permanent	
**tension**			***none***	partial (ears)	stupor					
**vocalization (sounds expressions)**			***none***	provoked	spontaneous	expressive				
**stereotypy**			***none***	head weaving	body weaving	grooming	circling	others		
**bizarre behavior**			***none***	head	body	self-mutilation	abnormal movements	others		
**approach response**				no reaction	***normal***	increased reaction	energetic reaction	exaggerated reaction		
**touch response**				no reaction	***normal***	increased reaction	energetic reaction	exaggerated reaction		
**click response**				no reaction	***normal***	increased reaction	energetic reaction	exaggerated reaction		
**tail-pinch response**				no reaction	***normal***	increased reaction	energetic reaction	exaggerated reaction		
**pupil size**	miosis	slight narrowing	***normal***	slight dilation	mydriasis					
**pupil response**			no reaction	slow reaction	***normal reaction***					
**air-righting reflex from the vertical position**				***normal***	slightly uncoordinated	lands on side	lands on back			
**respiration**	breathless	slow breathing	***normal***	accelerated breathing	stuffiness					
